# Retraction: Burkholderia cepacia Bacteremia Complicated by Acute Parotid Abscess in a Poorly Controlled Diabetic Patient With Community-Acquired Pneumonia: A Case Report

**DOI:** 10.7759/cureus.r221

**Published:** 2026-03-06

**Authors:** Nicholsan Jesiah, Pakkiyaretnam Mayurathan

**Affiliations:** 1 University Medical Unit, Teaching Hospital Batticaloa, Batticaloa, LKA; 2 Department of Clinical Sciences, Faculty of Health-Care Sciences, Eastern University of Sri Lanka, Batticaloa, LKA

This article has been retracted by the Editor-in-Chief after the authors contacted the journal to request retraction, stating that they misidentified the bacteria as burkholderia cepacia. Per the authors, confirmatory testing conducted after publication identified the bacteria as burkholderia pseudomallei. As a result, this article can no longer be considered accurate or credible and must be retracted.

